# Evaluation of plan quality and treatment efficiency for single‐isocenter/two‐lesion lung stereotactic body radiation therapy

**DOI:** 10.1002/acm2.12500

**Published:** 2018-12-08

**Authors:** Lana Sanford, Janelle Molloy, Sameera Kumar, Marcus Randall, Ronald McGarry, Damodar Pokhrel

**Affiliations:** ^1^ Department of Radiation Medicine University of Kentucky Lexington KY USA

**Keywords:** lung cancer, SBRT, single‐isocenter/two‐lesion, VMAT

## Abstract

**Purpose/objectives:**

To evaluate the plan quality and treatment delivery efficiency of single‐isocenter/two‐lesions volumetric modulated arc therapy (VMAT) lung stereotactic body radiation therapy (SBRT).

**Materials/methods:**

Eight consecutive patients with two peripherally located early stage nonsmall‐cell‐lung cancer (NSCLC) lung lesions underwent single‐isocenter highly conformal noncoplanar VMAT SBRT treatment in our institution. A single‐isocenter was placed between the two lesions. Doses were 54 or 50 Gy in 3 and 5 fractions respectively. Patients were treated every other day. Plans were calculated in Eclipse with AcurosXB algorithm and normalized to at least 95% of the planning target volume (PTV) receiving 100% of the prescribed dose. For comparison, two‐isocenter plans (isocenter placed centrally in each target) were retrospectively created. Conformity indices (CIs), heterogeneity index (HI), gradient index (GI), gradient distance (GD), and D_2cm_ were calculated. The normal lung V5, V10, V20, mean lung dose (MLD) and other organs at risk (OARs) doses were evaluated. Total number of monitor units (MUs), beam‐on time, and patient‐specific quality assurance (QA) results were recorded.

**Results:**

The mean isocenter to tumor distance was 6.7 ± 2.3 cm. The mean combined PTV was 44.0 ± 23.4 cc. There was no clinically significant difference in CI, HI, GD, GI, D_2cm_, and V20 including most of the OARs between single‐isocenter and two‐isocenter lung SBRT plans, evaluated per RTOG guidelines. However, for single‐isocenter plans as the distance between the lesions increased, the V5, V10, and MLD increased, marginally. The total number of MUs and beam‐on time was reduced by a factor of 1.5 for a single‐isocenter plan compared to a two‐isocenter plan. The single‐isocenter/two‐lesions VMAT lung SBRT QA plans demonstrated an accurate dose delivery of 98.1 ± 3.2% for clinical gamma passing rate of 3%/3 mm.

**Conclusion:**

The SBRT treatment of two peripherally located lung lesions with a centrally placed single‐isocenter was dosimetrically equivalent to two‐isocenter plans. Faster treatment delivery for single‐isocenter treatment can improve patient compliance and reduce the amount of intrafraction motion errors for well‐suited patients.

## 1 INTRODUCTION

For medically inoperable stage I/II nonsmall‐cell lung cancer (NSCLC) patients, several Phase I/II trials have shown that the use of stereotactic body radiation therapy (SBRT) treatment for solitary lung lesions representing the primary tumor mass is safe, effective, and has a high cure rate comparable to surgery.^1^, ^2^, ^3^, ^4^, ^5^, ^6^, ^7^ In these studies, medically inoperable patients with early‐stage NSCLC who underwent SBRT had 3‐yr primary tumor local control rates of up to 98% and a low risk of treatment‐related toxicity.

In the setting of either multiple primary lung cancers or limited metastatic lesions to the lungs (oligometastastic), SBRT presents a relatively new treatment opportunity. Optimal treatment planning must consider microscopic disease extension around the visible mass and allow for tumor movement, primarily due to respiration. Multiple metachronous or synchronous lung cancers are relatively common and have been managed by SBRT.^8^ Based on Phase I/II trials of SBRT in the management of oligometastastic lung lesions, for patients with one to three tumors, up to five tumors (with curative intent) and more than five tumors with palliative treatment have been reported.^9^, ^10^ Rusthoven and colleagues treated 38 patients, 63 total tumors, with lung SBRT of total dose of 48–60 Gy in 3 fractions. Actuarial local control rates at 1‐ and 2‐yr after SBRT was 100% and 96% respectively.^10^

SBRT to multiple lung lesions presents with technical challenges and can be treated either sequentially with separate treatment plans or synchronously to all lesions. However, the location and geometry of synchronous plans can be challenging since minor inaccuracies of patient setup can result in geometric misses. Attention must be paid to overlapping doses to organs at risk (OARs) and respiratory control is critical since different parts of the lung can move independently. Sequential treatment plans for each individual tumor, using a multi‐isocentric technique requires relatively longer planning and treatment delivery time. Safe and effective delivery of SBRT of lung requires precise, highly conformal treatment planning and delivery techniques.^11^, ^12^, ^13^ In the past decades, treatment techniques for lung SBRT included Linear accelerator‐based 3D‐conformal radiation therapy, intensity modulated radiation therapy (IMRT), volumetric modulated arc therapy (VMAT) (RapidArc,Varian Inc., Palo Alto, CA, USA), CyberKnife, and helical Tomotherapy (Accuray Inc., Sunnyvale, CA, USA). However, as the complexity of the technology has evolved, treatment has required very high total monitor units (MU) and relatively long treatment times to deliver a highly conformal plan and spare OARs.^14^, ^15^, ^16^

With the recent technological advances, VMAT may provide highly conformal radiation dose delivery with faster delivery times.^17^, ^18^, ^19^, ^20^ The VMAT lung SBRT simultaneously optimizes gantry speed, multileaf collimator (MLC) position and high dose‐rate (FFF, flattening filter free mode) to provide highly conformal dose distributions to the planning target volume (PTV) while minimizing dose to adjacent OARs. Reducing treatment time would improve patient compliance which helps reduce error due to motion, and promote more efficient clinic flow. For multiple brain metastases, recent studies have shown that single‐isocenter VMAT can provide highly conformal radiosurgical dose distributions, excellent plan quality and safe and faster treatment delivery compared to conventional multi‐isocenter technique.^21^, ^22^, ^23^ However, there is little literature in the medical physics community on the treatment of multiple lung lesions using single‐isocenter VMAT‐SBRT technique.

A few studies have examined the use of single‐isocenter SBRT for multiple lung lesions. A study by Trager et al.^24^ discusses the use of a technique that utilizes a single‐isocenter with distinct optimizations for extracranial radiosurgery. Gulam et al.^25^ examined six patients and found that the criteria set forth by Radiation Therapy Oncology Group (RTOG) study 0915 protocol was met with regard to CI, but not some other critical dosimetric parameters. A retrospective study in total eleven patients by Quan et al.^26^ showed no difference in multiple dosimetric parameters between single‐isocenter VMAT plans (four single‐isocenter VMAT plans were compared) and multi‐isocenter intensity‐modulated SBRT to the lung. Still, the ability of a single‐isocenter treatment to two or more lung lesions to deliver curative treatment plans in adherence with RTOG 0915 dosimetric compliance criteria has not been fully explored. In this report we present our recently adopted treatment method utilizing single‐isocenter VMAT plan for SBRT of two lung lesions evaluated per RTOG 0915. For completeness, the original single‐isocenter lung SBRT plans and retrospectively generated conventional two‐isocenter lung SBRT plans were compared via their protocol compliance, plan quality, dose to critical structures, treatment delivery efficiency, and accuracy.

## 2 MATERIALS AND METHODS

### 2.A Patient setup and target delineation

A total of eight sequential patients were included in this retrospective study, all of whom had two peripherally located Stage I NSCLC lesions. The patients were immobilized using Body Pro‐Lok^™^ platform (CIVCO system, Orange City, IA) in the supine position with their arms above their head with abdominal compression, potentially reducing diaphragmatic motion to less than or equal to 1.0 cm. Conventional 3D CT scans and respiration‐correlated 4D CT scans were acquired on a GE Lightspeed 16 slice CT scanner (General Electric Medical Systems, Waukesha, WI) with 512 × 512 pixels at 2.5 mm slice thickness in the axial cine mode. Varian's Real Time Position Management Respiratory Gating System (version 1.7) was used for collection of 4D CT data. All 10 phases of 4D CT slices and respiratory motion signal were transferred to an Advantage 4D Workstation (General Electric Medical Systems, San Francisco, CA), where the maximum intensity projection (MIP) images were generated after a phase binning of the 4D CT images. In addition to the MIP images, the motion of both tumors was evaluated by an experienced physicist to affirm synchronous tumor motion that was less than 1 cm. The regular 3D CT scan and the MIP images were imported into the Eclipse treatment planning system (TPS) (version 13.0, Varian Medical Systems, Palo Alto, CA) and coregistered for target contouring. Gross tumor volumes (GTV) and internal tumor volumes (ITV) were delineated on the 3D CT images with references to the MIP images. Planning target volumes (PTV) were generated by adding non‐uniform 5–10 mm margins to the ITV to accommodate the patient setup uncertainties based on tumor size, location and synchronous tumor motion. The critical structures, such as bilateral lungs excluding the ITV (normal lung), spinal cord, ribs, heart, great vessels, esophagus, and skin were delineated on the 3D CT images.

### 2.B Treatment planning

#### 2.B.1 Clinical single‐isocenter VMAT Plan

Highly conformal, clinically optimal VMAT treatment plans were generated using 3–4 non‐coplanar partial arcs (5–10°, couch kicks were used for arcs) for the Truebeam linear accelerator (Varian, Palo Alto, CA) with millennium MLC and a 6 MV‐FFF (1400 MU/min) beam. A single‐isocenter was placed approximately between the two lesions. As the isocenter location does not need to be exactly in the middle of the lesions, an offset allowing for the gantry to rotate in a partial arc can be made. For those arcs, collimator angles were chosen in such a way that the opening of the MLC between tumors was minimized while the gantry rotates around the patient. Additionally, jaw tracking was used to further minimize the out of field leakage dose. The isocenter to tumors distance was the maximum 3D‐linear distance from the single‐isocenter location to the geometric center of the individual tumor/isocenter. This distance was calculated in the TPS using the x‐, y‐, and z‐ primary coordinates of the tumor centers. This distance was estimated to evaluate the normal lung doses as a function of isocenter distance from the targets. A dose of 54 or 50 Gy in 3 and 5 fractions was prescribed to the PTV D95%. All clinical treatment plans were calculated using the Eclipse TPS with Acuros‐XB (version 13.6.0, Varian Medical Systems, Palo Alto, CA) algorithm on the 3D CT images for heterogeneity corrections with a 2.0 × 2.0 × 2.0 mm^3^ dose calculation grid‐size. Dose to medium reporting mode was selected. All clinical plans were inversely optimized using variation of gantry rotation speed, dose rate and MLC positions. The generalized normal tissue objective (NTO) parameters were used to control the gradients for single‐isocenter clinical plan. As recommended by Varian, in our department, we used the following NTO parameters for lung SBRT plans: NTO with high priority of 150 with distance to target border of 0.1 cm. Start dose of 100.0% and end dose of 40% was used with a fall‐off factor of 0.5/mm. Moreover, the ring structures of 5, 10, and 20 annulus from each lesion with 5 mm gaps were generated to enforce the high dose regions (typically enforcing maximum 120% hotspot inside each ITV) and minimize the intermediate dose spillage. All the planning objectives were per RTOG 0915 guidelines. The patients were treated every other day per lung SBRT protocol.

#### 2.B.2 Two‐isocenter VMAT plan

For comparison, the SBRT treatment plans for all patients were retrospectively replanned with a conventional two‐isocenter approach. Individual isocenters were placed in the geometric center of each tumor. For each target, the plans were generated using 3–4 noncoplanar partial arcs, similar to single‐isocenter plan. Collimator rotations and jaw tracking were applied. The plan for the first tumor (PTV1) was first computed using same RTOG guidelines as described before. The plan for PTV1 was then used as the base‐plan for generating the plan for the second tumor (PTV2) in order to allow full scatter contributions from both plans. All the planning objectives used were the same as the single‐isocenter plan including the NTO parameters and ring structures. Dosimetric parameters for the target coverage and the adjacent OARs, including normal lung, were evaluated.

### 2.C Plan evaluation

Each plan was evaluated for the target coverage and the dose to OARs. For example, using the percentage prescribed isodose volume and target size, the RTOG conformity index (CI) was calculated as follows:^27^

(1) $$\mathrm{RTOG}\;\mathrm{CI}=\frac{\mathrm{Rx}\;\mathrm{Isodose}\;\mathrm{Volume}}{\mathrm{PTV}\;\mathrm{volume}}$$

Ideally, CI = 1.0, implying a perfectly conformal plan. The RTOG recommendation for the CI is <1.2 with 1.2–1.5 being acceptable with minor deviations. In addition, the Paddick conformation number (CN) was calculated by:^28^

(2) $$\mathrm{Paddick}\;\mathrm{CN}=\frac{{\left({\mathrm{TV}}_{\mathrm{PIV}}\right)}^{2}}{\left(\mathrm{TV}*\mathrm{PIV}\right)}$$

where TV_PIV_ is the target volume covered by the prescription isodose volume, TV is the target volume and PIV is the prescription isodose volume. CN = 1.0 would be ideal. The heterogeneity index (HI) was determined by:

(3) $$\mathrm{HI}=\frac{D10\%}{D95\%}$$

where D10% is the dose to the hottest 10% of the PTV and D95% is the dose to the 95% of the PTV coverage. The intermediate dose spillage was evaluated by using, gradient index (GI), D_2cm_ and gradient distance (GD). The GI was given by:

(4) $$\mathrm{GI}=\frac{R50\%}{R100\%}$$

where R50% is the ratio of 50% prescription isodose volume to the PTV and R100% is the ratio of 100% prescription isodose volume to the PTV. Per RTOG, depending on the target size, a GI of 3.0–6.0 is desirable. Similarly, D_2cm_ is the maximum dose, in percent of dose prescribed, at 2 cm from the PTV in any direction; and the GD, is the average distance from 100% prescription dose to 50% of the prescription dose. Although, RTOG only recommended normal lung, V20 < 10% (10–15% was acceptable with minor deviations), we have evaluated V5, V10, and mean lung dose (MLD) for normal lung for all plans.

### 2.D Dose to other OARs

In addition to the lung dose, all the clinical single‐isocenter plans were evaluated for dose to spinal cord, heart, esophagus, trachea, ribs, and skin per RTOG guidelines. The dose volume histogram parameters were compared between the single‐isocenter and the two‐isocenter plans. The mean and standard deviation values for each of the dose metrics were compared using paired *t* tests for single‐isocenter vs two‐isocenter computed dosimetric parameters for the OARs dose tolerances using an upper bound of *P* < 0.05.

### 2.E Delivery efficiency and accuracy

The dose delivery efficiency of each lung SBRT plan was evaluated based on total number of MU and actual beam‐on time. For the single‐isocenter plan, actual beam on time was recorded at the treatment machine while delivering the VMAT‐SBRT QA plan. Delivery accuracy of the VMAT‐SBRT QA plan was evaluated by physically measuring the 2D dose distribution of each plan using an Octavius phantom (PTW, Freiburg, Germany). All QA plans were delivered at the machine the day before the patient's 1st treatment. The measured cumulative 2D dose plan was compared with the computed dose distributions calculated on the Octavius QA phantom plan by the TPS. Upon completion of delivered dose, data were analyzed with Octavius MEPHYSTO Navigator (VeriSoft Patient Plan Verification, Version 6.3, PTW) using the standard clinical gamma passing rate criteria of 3%/3 mm maximum dose difference and distance‐to‐agreement (DTA) with 10% threshold as well as point dose. Since the two‐isocenter plans were not used for patient treatment, no VMAT QA was done. The beam on time was estimated by using dose rates of 1400 MU/min for these plans.

## 3 RESULTS

### 3.A Target coverage and normal lung dose

**Figure 1:**
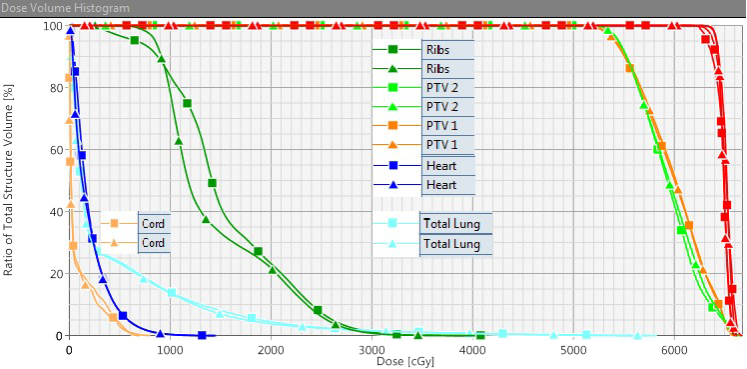
This shows the dose volume histogram comparison for the target coverage (for both PTV1 and PTV2). The ITVs (red) and a few OAR such as total normal lung (light blue), heart (dark blue), ribs (green), and spinal cord (orange) are shown for patient #8. Prescription dose was 54 Gy in three fractions. The square symbols representing the single‐isocenter plan, and the triangle symbols representing the two‐isocenter plan. Both plans were normalized to at least 95% of PTV received 100% of the prescribed dose. In this case, the isocenter to tumors distance was about 4 cm; the dosimetrically equivalent plans were generated using single‐isocenter technique, as demonstrated, with similar target coverage and dose to the OARs.

All patients were treated with a single‐isocenter VMAT plan in our clinic, which utilized 2–4 noncoplanar partial arcs. The prescription dose was 50–54 Gy in 3–5 fractions for at least 95% of the PTV receiving 100% of the prescribed dose. The single‐isocenter to tumors distance was calculated in the TPS using the x‐, y‐, and z‐ primary coordinates of the tumor centers, as described above. The isocenter to tumor distance was approximately 3.7 to 9.6 cm (mean, 6.7 ± 2.3 cm). The mean combined PTV was 44.0 ± 23.4 cc (range, 20.5–91.8 cc). The DVHs for both single‐isocenter and two‐isocenter treatment plans are shown in Fig. 1 for patient #8. In this case, both planning approaches produced dosimetrically equivalent plans. However, the treatment delivery time for the single‐isocenter technique is reduced by a factor of 1.5. That was just a reported treatment delivery time, the actual patient setup and verification for the second isocenter with two‐isocenter plan would take extra‐time, prolonging the treatment delivery.

**Figure 2:**
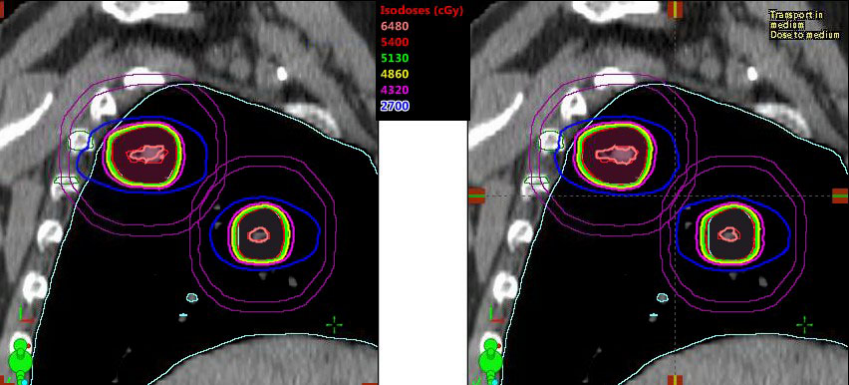
This is a comparison of isodose distributions in sagittal view for the same patient #8 generated via single‐isocenter and two‐isocenter plans. In the right panel a single‐isocenter location is shown by the intersection of the cross‐hair; in the left panel two‐isocenter plan sum is shown for the both targets (PTV1 and PTV2). Target volumes contoured include both ITVs (red, innermost) followed by PTVs (orange and green, outermost). Higher isodose lines, such as 54 Gy (100%), 51.3 Gy (95%), 48.6 Gy (90%), 43.2 Gy (80%), exhibit sharp dose fall off for the both plans, including 27.0 Gy (50%) isodose line (blue). In both plans, the hotspot, 120% isodose line (thick‐orange) was shown in the middle of the ITV. Other OARs such as ribs and lung contours are shown. Purple color rings were contoured to calculate D_2cm_ (%) for each target.

Figure 2 displays a sagittal view of both single‐isocenter and two‐isocenter treatment plans for the same patient (#8). In this case, the normal lung V5 and V10 were similar; V20 was slightly higher with single‐isocenter plan compared to two‐isocenter plan. However, both plans met the RTOG compliance criteria for the target coverage (see Table 1), normal lung and the other OARs dose tolerances.

**Table 1:** Comparison of plan evaluation parameters for single‐isocenter vs two‐isocenter treatment plans of all eight lung SBRT patients. Lesion 1 (PTV1 plan) and Lesion 2 (PTV2 plan) and two‐isocenter (Two‐iso) plan sum

Patient no.	Plan type and tumor location	Combined PTV (cc)	RTOG CI	Paddick CN	HI	GI	D_2cm_ (%)	GD (cm)	Isocenter to tumors distance (cm)
1	Lesion 1, LUL	5.0	1.08	0.75	1.16	6.6	47.9	0.90	5.2
	Lesion 2, LLL	16.1	1.01	0.84	1.17	4.1	41.4	0.95	
	Two‐iso (plan sum)	21.1	1.05	0.81	1.18	4.8	47.6	0.97	
	Single‐isocenter		1.05	0.79	1.16	5.0	56.5	1.20	
2	Lesion 1, LUL	30.7	1.01	0.83	1.11	4.2	57.3	1.21	9.5
	Lesion 2, RUL	43.6	0.99	0.84	1.22	3.6	55.2	1.18	
	Two‐iso (plan sum)	74.3	1.02	0.80	1.23	4.2	60.2	1.24	
	Single‐isocenter		1.02	0.82	1.21	4.6	62.8	1.75	
3	Lesion 1, LLL	16.2	1.05	0.76	1.17	4.8	50.8	1.05	9.6
	Lesion 2, RUL	34.9	1.19	0.68	1.08	5.5	69.2	1.43	
	Two‐iso (plan sum)	51.1	1.26	0.70	1.26	5.4	76.3	1.49	
	Single‐isocenter		1.29	0.67	1.39	6.4	80.5	1.78	
4	Lesion 1, LLL	8.6	1.03	0.80	1.17	4.8	43.2	0.87	4.6
	Lesion 2, RUL	26.6	1.01	0.84	1.20	4.1	51.8	1.11	
	Two‐iso (plan sum)	35.2	1.07	0.76	1.22	4.9	55.9	1.21	
	Single‐isocenter		1.16	0.74	1.18	5.5	53.6	1.45	
5	Lesion 1, LUL	80.9	0.99	0.83	1.15	3.3	56.1	1.34	8.4
	Lesion 2, RLL	10.9	1.02	0.72	1.21	5.0	48.7	0.97	
	Two‐iso (plan sum)	91.8	1.01	0.81	1.17	3.9	57.6	1.38	
	Single‐isocenter		1.02	0.81	1.16	4.1	56.4	1.68	
6	Lesion 1, Ant. LLL	19.6	1.04	0.77	1.16	4.3	49.3	1.02	4.8
	Lesion 2, Post. LLL	7.7	1.20	0.63	1.20	6.7	44.8	1.00	
	Two‐iso (plan sum)	27.3	1.09	0.72	1.19	5.6	50.3	1.11	
	Single‐isocenter		1.03	0.76	1.17	5.3	48.7	1.38	
7	Lesion 1, RUL	13.6	1.04	0.67	1.10	5.3	48.0	1.04	4.9
	Lesion 2, LUL	17.2	1.02	0.78	1.05	4.5	48.6	1.03	
	Two‐iso (plan sum)	30.8	1.05	0.62	1.11	5.6	51.4	1.09	
	Single‐isocenter		1.04	0.70	1.16	5.2	48.6	1.43	
8	Lesion 1, Post. RUL	13.5	0.99	0.83	1.19	4.3	46.5	0.94	3.7
	Lesion 2, Ant. RUL	8.0	1.00	0.80	1.18	5.1	45.4	0.90	
	Two‐iso (plan sum)	21.5	1.04	0.81	1.19	4.8	47.0	1.13	
	Single‐isocenter		1.03	0.81	1.19	5.1	48.9	1.23	

Detail of the plan comparison for target coverage including tumor location and the tumors distance from the isocenter are shown in Table 1.

All lung SBRT plans were acceptable per RTOG guidelines for the high (CI, HI) and intermediate dose spillage (GI and D_2cm_). In addition, similar results were shown for the Paddick CN between the two plans. No clinically significant difference was observed in CI, HI, GD, GI, and D_2cm_ between single‐isocenter and two‐isocenter lung SBRT plans evaluated per RTOG guidelines by the treating physician. However, the GD values were slightly higher with single‐isocenter plan of about 3–5 mm, especially for the larger tumor distance from the isocenter compared to two‐isocenter plan. Clinical significance of higher GD values, compared to relatively faster delivery of single‐isocenter plan, may need to be explored.

The absolute differences between single‐isocenter and two‐isocenter plans for normal lung V20, V10, V5, and MLD were listed in the Table 2. All patients had V20 < 10–15% for both treatment plans. A statistically insignificant difference (*P* = 0.09) was found for the normal lung V20 between two plans. However, V10, V5, and MLD increases slightly with single‐isocenter plan compared to two‐isocenter plan, giving statistically significant differences (*P* = 0.03, 0.01 and 0.03 respectively). Statistically significant *P*‐values are highlighted in bold (see Table 2). Although, V10, V5, and MLD had shown statistically significant differences, the absolute differences were on the order of less than 0.8% for V20, 2.8% for V10 and 6.5% for V5) and less than 60 cGy for MLD, on average, therefore, we do not expect the differences would be clinically significant.

**Table 2:** Normal lung doses statistics between single‐isocenter and two‐isocenter plans for all eight lung SBRT patients. Data were presented as mean ± standard deviation (range) and *P*‐values

Plan type	V20 (%)	V10 (%)	V5 (%)	MLD (Gy)
Two‐isocenter	6.7 ± 2.7 (2.9 to 12.2)	18.2 ± 6.7 (7.2 to 29.9)	29.7 ± 10.4 (21.1 to 46.5)	5.4 ± 1.4 (3.3 to 8.2)
Single‐isocenter	7.5 ± 13.4 (3.2 to 13.5)	21.0 ± 8.9 (7.5 to 36.8)	36.1 ± 13.8 (18.2 to 61.7)	6.0 ± 1.8 (3.7 to 9.2)
*P*‐value	0.09	**0.03**	**0.01**	**0.03**

Statistically significant *P*‐values are highlighted in bold.

**Figure 3:**
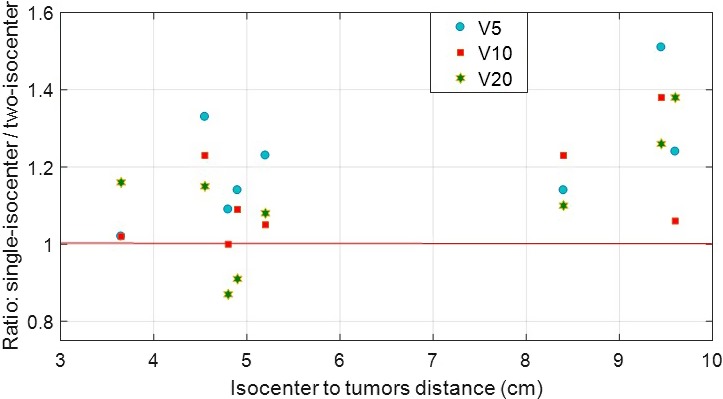
Scatter plot: For all eight lung SBRT patients, the ratios of V5, V10 and V20 of normal lung doses calculated by single‐isocenter and two‐isocenter plans as a function of isocenter to tumors distance. For the identical planning objectives, the single‐isocenter plan gave slightly higher values of V5, V10, and V20 by a factors of 1.2, 1.1, and 1.1, on average, respectively, compared to two‐isocenter plan. This suggests that comparable dosimetric parameters can be obtained for the normal lung. However, single‐isocenter plan would have considerably faster treatment delivery by an almost a factor of 2, eliminating the setup and verification time for the second isocenter plan.

The ratios between single‐isocenter and two‐isocenter plans for the V20, V10, and V5 as a function of isocenter to tumors distance can be seen in Fig. 3. When the isocenter to tumor distance increased, the low dose volume to the normal lung, such as V5 and V10, was slightly increased. However, two of eight patients had lower values of V20 with single‐isocenter plan.

### 3.B Dose to other OARs

A comparison of other OARs dosimetric parameters for single‐isocenter and two‐isocenter plans for all eight lung SBRT patients is presented in Table 3. Critical organs such as spinal cord (D_max_, and D_0.35cc_), heart (D_max_ and D_15cc_), esophagus (D_max_ and D_5cc_), trachea (D_max_ and D_4cc_), ribs (D_max_ and D_1cc_), and skin (D_max_ and D_10cc_) were evaluated per SBRT protocol guidelines.

**Table 3:** Average values of absolute dose differences between single‐isocenter and two‐isocenter plans for the other major dose distribution parameters of the OARs for all eight lung SBRT patients

OARs	Parameters	Mean ± SD (Gy)	Range (Gy)	Ratio^a^	*P*‐value
Spinal cord	D_max_	0.5 ± 1.1	−0.9 to 2.9	1.05 ± 0.13	0.25
	D_0.35cc_	0.5 ± 1.1	−0.7 to 2.7	1.03 ± 0.13	0.62
Heart	D_max_	0.9 ± 3.0	−5.4 to 5.0	1.07 ± 0.14	0.42
	D_15cc_	2.0 ± 1.2	0.0 to 3.9	1.15 ± 0.09	**0.002**
Esophagus	D_max_	2.1 ± 3.9	−4.5 to 3.5	1.13 ± 0.23	0.18
	D_5cc_	1.9 ± 3.3	−3.3 to 4.6	1.18 ± 0.31	0.15
Trachea	D_max_	0.7 ± 1.8	−5.0 to 5.9	1.13 ± 0.27	0.55
	D_4cc_	−0.8 ± 1.8	−4.5 to 1.0	0.96 ± 0.27	0.27
Ribs	D_max_	0.0 ± 3.9	−5.1 to 7.4	0.99 ± 0.08	0.98
	D_1cc_	−0.1 ± 2.2	−4.5 to 2.4	0.99 ± 0.07	0.91
Skin	D_max_	−0.6 ± 1.5	−3.9 to 0.6	0.97 ± 0.07	0.28
	D_10cc_	1.2 ± 1.1	−0.4 to 2.8	1.11 ± 0.08	**0.02**

Absolute dose differences = single‐isocenter–two‐isocenter. The negative sign indicates that the results of the two‐isocenter plans were larger than those of single‐isocenter plans. Statistically significant *P*‐values are highlighted in bold.

a Single‐isocenter/two‐isocenter.

The average values of maximum doses to spinal cord, ribs, and skin were similar (also see the average of the ratios in Table 3) between the two planning methods. Although, the average values of the absolute dose differences and ratios for heart, esophagus and trachea were slightly higher with single‐isocenter plan, the average absolute dose differences were up to 1–2 Gy. While evaluating those plans per SBRT protocol's guidelines, those values met the protocol criteria, therefore, the differences were not deemed clinically significant. Almost all *P*‐differences were insignificant, except for dose to 15 cc of heart (*P* = 0.002) and dose to 10 cc of ribs (*P* = 0.02). Both the single‐isocenter and two‐isocenter plans were within clinically acceptable limits per RTOG 0915.

### 3.C Delivery efficiency and accuracy

For single‐isocenter plans, the mean values of total number of MUs and beam on time were 6014 (4013 to 10,727) and 4.3 min (2.9 to 7.7 min). For each clinical single‐isocenter plan, actual beam‐on time was recorded at the treatment machine (to verify the calculated beam on time) while delivering VMAT QA plan as mentioned earlier. For all cases reported here, the maximum dose rate of 1400 MU/min for 6X‐FFF beam was used. That (dose rate) was reviewed for each VMAT arc for all patients under the MLC properties tab. In addition, maximum dose rate of 1400 MU/min was visually observed (all the time) at the Octavius VMAT QA delivery at Truebeam for all clinical single‐isocenter/two‐lesion lung SBRT plans. This suggest that for these high dose (high MUs) per fraction treatment the beam on time was dictated by total number of MUs per arc (as expected) rather than gantry rotation speed. Compared to two‐isocenter plans, the total number of MUs and beam on time were reduced by a factor of 1.5. Furthermore, with two‐isocenter plans, the actual patient setup and verification for the second isocenter plan would take extra‐time, prolonging the treatment delivery. In addition, lower total MUs could potentially deliver lower leakage dose. The complete details regarding number of MUs, beam‐on time, VMAT QA gamma pass rates, and the measured point dose percent difference are found in Table 4. Since the isocenter location for single‐isocenter was under the MLC, the maximum point dose was measured at the middle of the targets where the maximum fluence was delivered off axis to the two targets and compared to the computed VMAT QA plan on Octavius phantom.

**Table 4:** The detailed information on total number of MUs and beam‐on time for the both single‐isocenter and two‐isocenter plans for all eight lung SBRT patients. The Octavius VMAT‐SBRT QA pass rates and point dose measurements for single‐isocenter plans were also shown

Patient no.	Plan type	Total no. of MUs	Beam‐on time (min)	Gamma pass rates 3%/3mm (%)	Point dose % diff. (%)
1	Two‐isocenter	10,069	7.19	—	—
	Single‐isocenter	5777	4.13	99.3	0.9
2	Two‐isocenter	13,198	9.43	—	—
	Single‐isocenter	10,727	7.66	91.7	1.8
3	Two‐isocenter	9095	6.50	—	—
	Single‐isocenter	6607	4.72	100.0	1.5
4	Two‐isocenter	7185	5.13	—	—
	Single‐isocenter	6029	4.31	100.0	2.3
5	Two‐isocenter	6219	4.44	—	—
	Single‐isocenter	4093	2.92	99.4	0.3
6	Two‐isocenter	9047	6.46	—	—
	Single‐isocenter	5047	3.61	94.3	0.4
7	Two‐isocenter	5608	4.01	—	—
	Single‐isocenter	4149	2.96	100.0	0.4
8	Two‐isocenter	10,500	7.50	—	—
	Single‐isocenter	5680	4.06	100.0	0.7
Mean ± SD	Two‐isocenter	8865 ± 2330	6.3 ± 1.7	—	—
	Single‐isocenter	6014 ± 1963	4.3 ± 1.4	98.1 ± 3.0	1.04 ± 0.7

The Octavius VMAT QA pass rates for the single‐isocenter plan was 98.1 ± 3.0%, on average, for 3%/3 mm clinical gamma pass rate criteria and the point dose measurement was about within 1%, on average, suggesting that an accurate delivery of the lung SBRT plan. However, for patient #2, the gamma pass rates were around 92% for 3%/3 mm criteria. In this case, both tumors were relatively large, and the tumors to isocenter distance was relatively large, around 9.5 cm. In addition, the tumors were located in the bilateral lungs, therefore, the MLCs have to travel a longer distance, providing suboptimal VMAT QA pass rates; suggesting that exceeding 10 cm (isocenter to tumors distance) may not provide clinically optimal plan with single‐isocenter. While reanalyzing those data with a tighter distance‐to‐agreement (3%/2 mm) criteria, the average value of gamma pass rate was 95.8 ± 3.8% (ranged, 90.6 to 100%) that was within the departmental SBRT VMAT QA pass rate criteria (>/=90.0% pass rates). Since, the two‐isocenter plans were not used for actual patient's treatment we did not run VMAT QA for those plans.

## 4 DISCUSSION

In this study, we have presented our initial clinical experiences of a fast, effective, and accurate treatment planning and delivery technique using single‐isocenter VMAT plans for SBRT of two lung lesions following RTOG 0915 protocol guidelines.^12^ Our single‐isocenter VMAT plan for SBRT of two lung lesions uses 3–4 noncoplanar partial arcs with jaw tracking and patient specific collimator angles to minimize leakage dose from leaves travelling in between the tumors. Single‐isocenter VMAT‐SBRT plans were highly conformal and achieved adequate target coverage (see Table 1 for CI, HI, Paddick CN, GI, D_2cm_, and GD) compared to conventional two‐isocenter plans. For all patients, the single‐isocenter plans met RTOG guidelines including normal lung V20 and were similar compared to two‐isocenter plans. However, when the isocenter to tumors distance increased, the low dose volume to the normal lung, such as V5 and V10, was slightly increased as shown in Fig. 3. In addition, the other OARs such as spinal cord, heart, esophagus, trachea, ribs, and skin dose tolerances were also within protocol. The single‐isocenter treatment was well‐tolerated with all patients. The beam on time was 4.3 min and VMAT‐SBRT QA gamma passing rates were 98.1% (3%/3 mm clinical gamma passing criteria), on average, demonstrating an excellent potential for a fast, reliable, and accurate delivery of single‐isocenter VMAT lung SBRT treatment for two lung lesions.

The single‐isocenter plan for treating multiple lung tumors has been reported by a few investigators.^29^, ^30^ For instance, using both coplanar and noncoplanar nine fields IMRT (in Pinnacle TPS), Zhang et al.^29^ compared those IMRT plans with helical Tomotherapy for single‐isocenter/multitarget lung SBRT treatment. The prescription was 60 Gy in three fractions. In their study, it was concluded that compared to IMRT, helical Tomotherapy gave better target coverage at the cost of overall 73.0 ± 20.6 min treatment time. However, IMRT treatment time was not reported. It was also highlighted that compared to IMRT plans, Tomotherapy plan also gave a relatively higher normal lung V5. Another study by Li et al.^30^ reported that they treated two patients with single‐isocenter lung SBRT plan for more than five lung metastases lesions. Their prescription doses were 48 Gy/8 fractions for Patient A (5 tumors) and 42 Gy/7 fractions for patient B (7 tumors). Plans were generated in Monaco TPS (CMS Software Inc., St Louis, MO) using a few partial‐arcs and delivered with Elekta Axesse linear accelerator with 6 MV beam (660 MU/min). The beam on time for each treatment was about 10 min. Both patients were followed up, and the treatment was well‐tolerated by the patients with a minimal toxicity. In contrast, utilizing 6 MV‐FFF beam (in Eclipse) for Truebeam Linac our single‐isocenter VMAT planning technique delivered fast (average beam on time 4.3 min) and effective treatment (curative high biological effective dose of >100–150 Gy for each lesion) for relatively large cohorts of patients.

One potential concern for single‐isocenter VMAT‐SBRT plan for two lung lesions was low dose spill in the normal lung, such as V20, V10, and V5. Per RTOG recommendation, all our single‐isocenter/two‐lesions VMAT lung SBRT plans had V20 < 10–15%. Moreover, normal lung V5 was maintained less than 40%, on average.^31^, ^32^, ^33^ Although, in our experience when the isocenter to tumors distance increased, the normal lung V10 and V5 slightly increased, as expected, when compared to two‐isocenter plan. Our treatment planning strategy favored minimizing normal lung dose during single‐isocenter VMAT planning (by optimizing patient specific collimator angles in conjunction with jaws tracking such that the leakage dose due to the leaves travelling in between two tumors could be minimized) that could potentially help reduce severe lung toxicity with careful attention to V5 and V10 during plan optimization.

Another potential concern for single‐isocenter VMAT plan was the patient setup errors, for example tumor motion and rotational errors. This may result in geographic miss and compromise the local tumor control rates due to deformation. For single‐isocenter/two lesions VMAT plan isocenter was generally chosen at the midpoint of the two lesions, therefore, the isocenter distance between two lesions was evenly distributed. However, it would be difficult to find a perfect midpoint for noncoplanar lesions. The variability in respiratory patterns between the CT simulation and the time of treatment was studied by many researchers.^34^, ^35^, ^36^, ^37^ It has been reported in the literature that there were only small changes (within ±3 mm) due to intrafractional and interfractional motion while using conventional multi‐isocenter lung SBRT treatment. Their mean patient setup time from tumor localization to the end of treatment CBCT scan was about 40 min.^36^, ^37^ It was recommended that a 5 mm PTV margin was sufficient to address those motion errors. Furthermore, the spatial uncertainties for this kind of beam arrangement were discussed by Dr. Gary A Ezzell for single‐isocenter/multitarget cranial radiosurgery.^38^ In his paper it has been demonstrated that for Truebeam CBCT the maximum spatial uncertainties were less than 1.5 mm at 10 cm distance from the isocenter tested using 12 targets bearing balls (BBs) phantom. Before delivering each SBRT treatment, a daily quality assurance check on kilovoltage to megavoltage imaging isocenter coincidence was performed, including IsoCalc test for precise and accurate target localization. Our IsoCalc localization accuracy for Truebeam was <0.5 mm at isocenter. In addition, our off‐axis localization accuracy was similar to that of previously reported values by Dr. Ezzell while measured using an IsoCalc QA phantom embedded with the multiple BBs. All the quality assurance procedures were in compliance for SBRT treatment delivery. Our image guidance CBCT matching parameters (at Truebeam) were consistent with those previous finding.

For our single‐isocenter/two‐lesion lung SBRT treatment, in addition to abdominal compression, the synchronous tumor motion was captured at the 4D CT simulation and appropriate PTV margins were applied for each tumor using MIP images. Using single‐isocenter plan we have treated the PTV volume ranged from 5 to 90 cc (see Table 1). With this treatment technique, our clinical experience was that treating small tumors off axis would need an additional margin to minimize residual spatial uncertainties as mentioned above. On the other hand, treating larger tumors off axis may potentially spill low/intermediate dose to the normal lungs due to the MLC transmission. Our treating physicians are aware of these dosimetric characteristics and those residual spatial uncertainties were accounted during target delineation. However, our average beam on time of about 4.3 min per treatment could potentially decrease the possibility of changes on breathing signals from coughing or pain and making geographic miss unlikely‐potentially improving patient stability.

In addition, due to rotational errors, for small targets and those away from the single‐isocenter could potentially alter the dose distributions. For those highly conformal VMAT plans, the small deviation in motion error could potentially irradiate normal tissues, and it may increase the chance of radiation‐induced toxicity or miss the target. Our attending physician has addressed that issue by individually reviewing these target volumes and the associated tumor motion pattern and by assigning appropriate ITV to PTV margins (usually 5 mm in the medio‐lateral and anterior–posterior directions and 8 to 10 mm in superior–inferior direction) to accommodate potential tumor deformation. Moreover, great care has been taken by our treating physician and the physicist to address some of the above‐mentioned issues, for example, being available for the patient setup (in the 3D, 4D CT simulation and each treatment), image guidance, and CBCT matching and physically authorizing each treatment fraction for all patients. However, it is worthwhile to mention here that the 8–10 mm superior–inferior expansion of the ITV to PTV is not a requirement for an effective treatment of two lung lesions using a single‐isocenter plan, but this was really only a conservative preference of our treating physicians from their many years of lung SBRT experiences. Further studies are required to validate the standard 5 mm ITV to PTV expansions that would be adequate or not for this kind of treatment setting while fulfilling the RTOG compliance.

In summary, each plan was rigorously evaluated using the dosimetric parameters listed in the Tables 1, 2, and 3. All parameters were deemed acceptable for both single‐isocenter and two‐isocenter plans per SBRT protocol ‐ suggesting that single‐isocenter plan could be dosimetrically equivalent to two‐isocenter plan and a faster and equally effective treatment delivery which can be offered to well suited patients. In the future, these patients will be followed up clinically and evaluated for local control rates and treatment related toxicity such as the effect of normal lung dose as a function of isocenter to tumors distance. Moreover, single‐isocenter VMAT plan for SBRT of lung for more than two lesions will be investigated.

## 5 CONCLUSION

This report presents our initial clinical experience with a single‐isocenter for two‐lesion SBRT procedure for lung tumors and compared with conventional two‐isocenter plan. Treatment of peripherally located two lung lesions with centrally assigned single‐isocenter was dosimetrically equivalent to two‐isocenter plan. For single‐isocenter plans, it was observed that as the distance between the lesions increased the normal lung V5, V10 and MLD somewhat increased. The single‐isocenter technique was fast, accurate, and very well‐tolerated by all the patients, improving patient comfort and potentially reducing the amount of intrafraction motion errors for well‐suited patients. Clinical follow‐up of these patients is warranted to determine the tumor local control rates and treatment related toxicity.

## CONFLICT OF INTEREST

The authors declare no conflict of interest.

## References

[1] Timmerman R , Paulus R , Galvin J , et al. Stereotactic body radiation therapy for inoperable early stage lung cancer. JAMA. 2010;303:1070–1076. 20233825 10.1001/jama.2010.261 PMC2907644

[2] Senan S , Palma DA , Lagerwaard FJ . Stereotactic ablative radiotherapy for stage I NSCLC: recent advances and controversies. J Thorac Dis. 2011;3:189–196. 22263087 10.3978/j.issn.2072-1439.2011.05.03 PMC3256515

[3] McGarry RC , Papiez L , Williams M , et al. Stereotactic body radiotherapy of early‐stage non‐small‐cell lung carcinoma: phase I study. Int J Radiat Oncol Biol Phys. 2005;63:1010–1015. 16115740 10.1016/j.ijrobp.2005.03.073

[4] Fakiris AJ , McGarry RC , Yiannoutosis C , et al. Stereotactic body radiotherapy of early‐stage non‐small‐cell lung carcinoma: four‐year results of prospective phase II study. Int J Radiat Oncol Biol Phys. 2009;75:677–6782. 19251380 10.1016/j.ijrobp.2008.11.042

[5] Timmerman R , McGarry R , Yiannoutsos C , et al. Excessive toxicity when treating central tumors in a phase II study of stereotactic body radiation therapy for medically inoperable early‐stage lung cancer. J Clin Oncol. 2006;24:4833–4839. 17050868 10.1200/JCO.2006.07.5937

[6] Iyengar P , Westover K , Timmerman RD . Stereotactic ablative radiotherapy (SABR) for non‐small cell lung cancer. Semin Respir Crit Care Med. 2013;34:845–854. 24258574 10.1055/s-0033-1358554

[7] Onishi H , Shirato H , Nagata Y , et al. Stereotactic body radiotherapy (SBRT) for operable stage I non‐small‐cell lung cancer: can SBRT be comparable to surgery? Int J Radiat Oncol Biol Phys. 2011;81:1352–1358. 20638194 10.1016/j.ijrobp.2009.07.1751

[8] Sinha B , McGarry RC . Stereotactic body radiotherapy for bilateral primary lung cancers: the Indiana University experience. Int J Radiat Oncol Biol Phys. 2006;66:1120–1124. 17145532 10.1016/j.ijrobp.2006.06.042

[9] Okunieff P , Petersen AL , Phillip A , et al. Stereotactic body radiation therapy (SBRT) for lung metastases. Act Oncol. 2006;45:808–817. 10.1080/02841860600908954 16982544

[10] Rusthoven KE , Kavanagh BD , Burri SH , et al. Multi‐institutional phase I/II trial of stereotactic body radiation therapy for lung metastases. J Clin Oncol. 2009;27:1579–1584. 19255320 10.1200/JCO.2008.19.6386

[11] Benedict SH , Yenice KM , Followill D , et al. Stereotactic body radiation therapy: the report of AAPM Task Group 101. Med Phys. 2010;37:4078–4100. 20879569 10.1118/1.3438081

[12] A Randomized Phase II Study Comparing 2 Stereotactic Body Radiation Therapy (SBRT) Schedules For Medically Inoperable Patients with Stage I Peripheral Non‐Small Cell Lung Cancer; RTOG 0915; 2014 (1‐67).

[13] Al‐Hallaq HA , Chmura S , Salama JK , et al. Rational of technical requirements for NRG‐BR001: the first NCI‐sponsored trial of SBRT for the treatment of multiple metastases. Pract Radiat Oncol. 2016;6:e291–e298. 27345129 10.1016/j.prro.2016.05.004 PMC5099083

[14] Sterzing F , Welzel T , Sroka‐Perez G , et al. Reir‐radiation of multiple brain metastases with helical tomotherapy. A multifocal simultaneous integrated boost for eight or more lesions. Strahlenther Onkol. 2009;185:89–93. 19240994 10.1007/s00066-009-1971-2

[15] Gibbs IC , Loo BW . Cyberknife stereotactic ablative radiotherapy for lung tumors. Technol Cancer Res Treatm. 2010;9:589–596. 10.1177/153303461000900607 21070081

[16] Nagai A , Shibamoto Y , Yoshida M , et al. Safety and efficacy of intensity‐modulated stereotactic body radiotherapy using helical tomotherapy for lung cancer and lung metastasis. Biomed Res Int. 2014;2014:473173. 24995299 10.1155/2014/473173 PMC4065754

[17] Kannarunimit D , Descovich M , Garcia A , et al. Analysis of dose distribution and risk of pneumonitis in stereotactic body radiation therapy for centrally located lung tumors: a comparison of robotic radiosurgery, helical tomotherapy and volumetric modulated arc therapy. Technol Cancer Res Treat. 2015;14:49–60. 24325136 10.7785/tcrt.2012.500394

[18] Chan MK , Kwong DL , Law GM , et al. Dosimetric evaluation of four‐dimensional dose distributions of CyberKnife and volumetric‐modulated arc radiotherapy in stereotactic body lung radiotherapy. J Appl Clin Med Phys. 2013;14:4229. 23835388 10.1120/jacmp.v14i4.4229 PMC5714543

[19] Merrow CE , Wang IZ , Podgorsak MB . A dosimetric evaluation of VMAT for the treatment of non‐small cell lung cancer. J Appl Clin Med Phys. 2013;14:4110. 10.1120/jacmp.v14i1.4110 PMC5714051 23318374

[20] Navarria P , Ascolese AM , Mancosu P , et al. Volumetric modulated arc therapy with flattening filter free (FFF) beams for stereotactic body radiation therapy (SBRT) in patients with medically inoper‐able early stage non‐small cell lung cancer (NSCLC). Radiother Oncol. 2013;107:414–418. 23725859 10.1016/j.radonc.2013.04.016

[21] Clark GM , Popple RA , Young PE , Fiveash JB . Feasibility of single‐isocenter volumetric modulated arc radiosurgery for treatment of multiple brain metastases. Int J Radiat Oncol Biol Phys. 2010;76:296–302. 19836151 10.1016/j.ijrobp.2009.05.029

[22] Clark GM , Popple RA , Prendergast BM , et al. Plan quality and treatment planning technique for single isocenter cranial radiosurgery with volumetric modulated arc therapy. Pract Radiat Oncol. 2012;2:306–313. 24674169 10.1016/j.prro.2011.12.003

[23] Nath SK , Lawson JD , Simpson DR , et al. Single‐isocenter frameless intensity‐modulated stereotactic radiosurgery for simultaneous treatment of multiple brain metastases: clinical experience. Int J Radiat Oncol Biol Phys. 2010;78:91–97. 20096509 10.1016/j.ijrobp.2009.07.1726

[24] Trager M , Salama J , Yin F‐F , Adamson J . SBRT treatment of multiple extracranial oligometastases using a single isocenter with distinct optimizations. J Radiosurg SBRT. 2017;4:265–273. 29296451 PMC5658822

[25] Gulam M , Gopal A , Wen N , et al. Single isocenter lung SBRT for multiple PTV lesions. Int J Radiat Oncol Biol Phys. 2014;90:S910–S911.

[26] Quan K , Xu KM , Lalonde R , et al. Treatment plan technique and quality for single‐isocenter stereotactic ablative radiotherapy of multiple lung lesions with volumetric‐modulated arc therapy or intensity‐modulated radiosurgery. Front Oncol. 2015;5:1–9. 26500888 10.3389/fonc.2015.00213 PMC4594030

[27] International Commission on Radiation Units and Measurements (ICRU) . Prescribing, recording and reporting photon beam therapy. ICRU Report 62. (Supplement to ICRU Report 50). Bethesda, MD: ICRU Publications; 1999.

[28] Paddick I . A simple scoring ratio to index the conformity of radiosurgical treatment plans. J Neurosurg. 2000;93:219–222. 11143252 10.3171/jns.2000.93.supplement

[29] Zhang Y , Chen Y , Qiu J , Yang J . Dosimetric comparisons of lung SBRT with multiple metastases by two advanced planning systems. Int J Med Phys Clin Eng Radiat Oncol. 2014;3:252–261.

[30] Li Q , Mu J , Gu W , et al. Frameless stereotactic body radiation therapy for multiple lung metastases. J Appl Clin Med Phys. 2014;15:105–115. 10.1120/jacmp.v15i4.4737 PMC5875519 25207400

[31] Madani I , De Ruyck K , Goeminne H , et al. Predicting risk of radiation‐induced lung injury. J Thorac Oncol. 2007;2:864–874. 17805067 10.1097/JTO.0b013e318145b2c6

[32] Baker R , Han G , Sarangkasiri S , et al. Clinical and dosimetric predictors of radiation pneumonitis in a large series of patients treated with stereotactic body radiation therapy to the lung. Int J Radiat Oncol Biol Phys. 2013;85:190–195. 22929858 10.1016/j.ijrobp.2012.03.041

[33] Guckenberger M , Baier K , Polat B , et al. Dose‐response relationship for radiation‐induced pneumonitis after pulmonary stereotactic body radiotherapy. Radiother Oncol. 2010;97:65–70. 20605245 10.1016/j.radonc.2010.04.027

[34] Hugo G , Vargas C , Liang J , et al. Changes in the respiratory pattern during radiotherapy for cancer in the lung. Radiother Oncol. 2006;78:326–331. 16564592 10.1016/j.radonc.2006.02.015

[35] Bosmans G , van Baardwijk A , Dekker A , et al. Intra‐patient variability of tumor volume and tumor motion during conventionally fractionated radiotherapy for locally advanced non‐small‐cell lung cancer: a prospective clinical study. Int J Radiat Oncol Biol Phys. 2006;66:748–753. 17011450 10.1016/j.ijrobp.2006.05.022

[36] Bissonnette JP , Franks KN , Purdie TG , et al. Quantifying interfraction and intrafraction tumor motion in lung stereotactic body radiotherapy using respiration‐correlated cone beam computed tomography. Int J Radiat Oncol Biol Phys. 2009;75:688–695. 19395200 10.1016/j.ijrobp.2008.11.066

[37] Li W , Purdie TG , Taremi M , et al. Effect of immobilization and performance status on intrafraction motion for stereotactic lung radiotherapy: analysis of 133 patients. Int J Radiat Oncol Biol Phys. 2011;81:1568–1575. 21075559 10.1016/j.ijrobp.2010.09.035

[38] Ezzell GA . The spatial accuracy of two frameless, linear accelerator‐based systems for single‐isocenter, multitarget cranial radiosurgery. J Appl Clin Med Phys. 2016;14:37–43. 10.1002/acm2.12044 PMC5689957 28300379

